# Enhanced Humid Reliability of Organic Thermoelectrics via Crosslinking with Glycerol

**DOI:** 10.3390/nano9111591

**Published:** 2019-11-09

**Authors:** Jaeyun Kim, Jae Gyu Jang, Jeonghun Kwak, Jong-In Hong, Sung Hyun Kim

**Affiliations:** 1 School of Electrical and Computer Engineering, University of Seoul, Seoul 02504, Korea; kimjaeyun24@naver.com; 2 Department of Chemistry, Seoul National University, Seoul 08826, Korea; jangjaegyu@snu.ac.kr; 3 Department of Electrical and Computer Engineering, Inter-university Semiconductor Research Center, Seoul National University, Seoul 08826, Korea; 4 Department of Carbon Convergence Engineering, Wonkwang Univeristy, Iksan 54538, Korea

**Keywords:** organic thermoelectrics, cross-linking, humid reliability, glycerol

## Abstract

Poly(3,4-ethylenedioxythiophene):poly(4-styrenesulfonate) (PEDOT:PSS) has shown significant achievements in organic thermoelectrics (TEs) as an alternative for inorganic counterparts. However, PEDOT:PSS films have limited practical applications because their performance is sensitive to humidity. Crosslinking additives are utilized to improve the reliability of PEDOT:PSS film through enhancing hydrophobicity; among these, polyethylene glycol (PEG) is a widely-used additive. However, ether groups in PEG induce water molecules in the film through the hydrogen bond, which deteriorates the TE reliability. Here, we enhance the TE reliability of the PEDOT:PSS film using glycerol as an additive through the crosslinking reaction between the hydroxyl group in glycerol and the sulfonic acid in PEDOT:PSS. The TE reliability (1/Power factor (PF)) of PEG solution-treated PEDOT:PSS film (PEG solution-treated film) was 57% of its initial absolute value (0 h), after 288 h (two weeks) in a humid environment (95% relative humidity, 27 °C temperature). On the other hand, the glycerol solution-treated PEDOT:PSS film (glycerol solution-treated film) exhibited superior TE reliability and preserved 75% of its initial 1/PF. Furthermore, glycerol vapor treatment enabled the film to have stronger TE humid reliability, maintaining 82% of its initial 1/PF, with the same condition. This enhancement is attributed to the increased hydrophobicity and lower oxygen content of the glycerol vapor-treated PEDOT:PSS film (glycerol vapor-treated film), which provides little change in the chemical composition of PEDOT:PSS.

## 1. Introduction

Thermoelectric (TE) generation, which can directly convert waste heat into electricity, has been developed as a solution to the global energy problem. In particular, organic TE materials have been attracting considerable attention due to their relative advantages compared to inorganic counterparts, such as low thermal conductivity (κ), and easy processing and synthesis. To date, many researchers have focused on enhancing electrical conductivity (σ) in the field of organic TE materials, which should yield a higher value for superior TE performance [1]. In this regard, most progress on organic TE performance has been achieved with poly(3,4-ethylenedioxythiophene):poly(4-styrenesulfonate) (PEDOT:PSS), for which an outstanding σ could be achieved through post-treatments with strong acid [2] or a high boiling point solvent [3]. However, organic TE films need to satisfy reliability for meeting commercial applications [4]. TE efficiency is evaluated by the dimensionless figure of merit, ZT = S^2^σT/κ, where S, T, σ, and κ are the Seebeck coefficient, absolute temperature, and electrical and thermal conductivity, respectively. The power factor (PF = S^2^σ) sometimes replaces ZT when it is difficult to evaluate κ in a thin film. Although the PF of PEDOT:PSS increases in a humid environment until film degradation, such the environment will worsen the TE stability with short exposure. Therefore, the reliability of organic TE film is necessary for successful energy harvesting for commercial TE applications.

In order to ensure long-term stability of σ for PEDOT:PSS film in a humid environment, many researchers have added a small number of crosslinking agents into PEDOT:PSS solution, which is a well-known approach [5,6,7,8,9,10,11,12]. Polymer crosslinking agents (e.g., polyethylene glycol (PEG) and polyvinyl alcohol (PVA)) also improve the stability of such devices by making a covalent chemical bond with the matrix polymer [5,8,9,10,11] The organic electronic devices with PEG solution-treated PEDOT:PSS film, however, have a trade-off relationship between the device performance and stability [13,14]. Moreover, the remaining oxygen atoms in the resultant film limit the enhancement of stability [13,14]. Due to this correlation, there is hesitation to use a polymer crosslinking agent for reliable organic TE films. Recently, we reported a reliable organic TE film by adding a small organic molecule into the PEDOT:PSS solution as a hydrophilic crosslinking agent, thereby enhancing both the PF and TE reliability through morphological/structural evolution [15]. We deduced that a small organic molecule is an appropriate alternative for a crosslinking additive to enhance the humid TE reliability. Among the candidates, we choose a glycerol to facilitate the PEDOT:PSS film with stronger reliability in the form of high boiling point, three hydroxyl groups, and hydrophilicity, which meet the prerequisites for a crosslinking reaction with high performance in the resultant film [6,16]. Although there are oxygen atoms in glycerol, the oxygen atoms are almost eliminated during the condensation reaction. Therefore, we can fabricate PEDOT:PSS TE film that is reliable in humidity. Additional advantages of glycerol in the fabrication of highly reliable PEDOT:PSS TE films are its eco-friendliness and recyclability. Thus, the liquid has the potential to serve as a green solvent for a chemical reaction [17,18,19]

Herein, to develop a PEDOT:PSS TE film that is reliable in humidity, we enhanced the hydrophobicity of the film through a crosslinking reaction between the hydroxyl group in glycerol and the sulfonic acid in PEDOT:PSS. The glycerol solution-treated PEDOT:PSS film (denoted glycerol solution-treated film) exhibited improved TE reliability (1/PF) with a retention of 75% of initial absolute value (0 h), while that of PEG solution-treated PEDOT:PSS film (denoted PEG solution-treated film) worsened to 57% of its initial absolute value, after 288 h (two weeks) storage in a humid environment (95% relative humidity, 27 °C temperature). Furthermore, the glycerol vapor treatment led to little change in the chemical composition of PEDOT:PSS under humid conditions compared to the glycerol solution treatment, which can be attributed to the lower oxygen content and more crosslinking linkages in the film. As a result, the glycerol vapor-treated film showed superior TE humid reliability and preserved 82% of its initial 1/PF value. In addition, the relationship between elastic modulus and κ allowed us to obtain the ZT value of 1.15 × 10^−2^ at 300 K for glycerol vapor-treated film, which was higher than for the PEG solution-treated film (ZT ≈ 0.74 × 10^−2^).

## 2. Materials and Methods 

### 2.1. Chemical Compounds

The PEDOT:PSS solution (Clevios PH 1000) with a concentration of 1.3 vol% dispersed in water was purchased from H. C. Starck GmbH (Leverkusen, Germany). Dimethyl sulfoxide (DMSO), glycerol, and poly(ethylene glycol) (PEG, average molecular weight: 200) were obtained from Sigma Aldrich (St. Louis, MO, USA). All materials were used as received.

### 2.2. Film Preparation

The glass substrates (25 mm × 25 mm) were cleaned with ultrasonic treatment in both distilled water and isopropyl alcohol, followed by drying in a vacuum oven for 12 h. As a control, the PEDOT:PSS film (Pristine film) was prepared as follows: PEDOT:PSS was mixed with 5 vol% DMSO and stirred for 30 min. The PEDOT:PSS with 5 vol% DMSO solution was filtered through a 0.45 μm pore size hydrophilic filter. A quantity of 2 vol% PEG or glycerol was added into the PEDOT:PSS solution with 5 vol% DMSO and stirred for 30 min, as reported in a previous procedure [13]. The cleaned glass substrates were treated with UV/ozone for 15 min, and the formulation solution was spin-coated at 2300 rpm for 40 s. The films were annealed at 150 °C for 30 min. PEG and glycerol solution treatments with Pristine film are denoted PEG solution-treated and glycerol solution-treated film, respectively. We placed Pristine film with glycerol- or PEG-treated solution (2 mL in a glass petri dish) inside a pre-heated vacuum chamber with the temperature at 150 °C for 15 min. A low vacuum state (10^−2^ Pa) was kept to maintain PEG or glycerol vapor atmosphere. The PEG and glycerol vapor treatments with Pristine film are denoted PEG and glycerol vapor-treated film, respectively.

### 2.3. Instruments and Sample Characterization

The thickness of the films was acquired using an alpha-step profilometer (Dektak Stylus Profilometer, Bruker, Billerica, MA, USA). The elastic modulus of each film was gained by measuring eight different locations on the films using an AFM (atomic force microscope, Bruker, Billerica, MA, USA) nanoindentation tip (NICT-MTAP). Fourier-transform infrared (FTIR) spectra were recorded with a PerkinElmer Frontier FT-NIR/MIR Spectrometer (PerkinElmer Inc., Waltham, MA, USA). The X-ray photoelectron spectroscopy (XPS) spectra were acquired from five different samples for each film using the SIGMA PROBE Model (Thermo VG Scientific, East Grinstead, UK). The contact angle of water on the films was measured by using a contact angle analyzer (Phoenix-10, Surface Electro Optics Co., Suwon City, Korea). The contact angles of the three films were taken by measuring four different locations.

### 2.4. TE Film Fabrication and Characterization

To fabricate a TE film, Au electrodes of 70 nm were thermally evaporated on a prepared film, through a shadow mask under a base pressure of <10^−4^ Pa. The length and width of the two electrodes were 17 mm and 8 mm, respectively, which were separated by 2 mm, as shown in Figure S1a. Two type-K thermocouples (Extech, EA15, Taipei, Taiwan) and current-voltage (I-V) probes were attached to the Au electrode at the same distance (10 mm distance spacing), respectively (Figure S1b). S was calculated from the equation S = ∆V/∆T, where ∆V and ∆T are the thermovoltage and the temperature difference between the cool and hot side of the film, respectively. The ∆V and ∆T were measured using an Agilent 3458A digital multimeter (Agilent Technologies Inc., Santa Clara, CA, USA, input impedance >10 GΩ) and an Extech EA15, respectively. All of the instruments were connected to a computer and controlled by LabView software (Version 2017, National Instruments, Austin, TX, USA). In order to verify the accuracy of our measurement setup, we measured the absolute Seebeck coefficient of pure nickel foil and obtained reasonable values of −20.2 ± 0.4 μV/K. The κ of glycerol-treated- and PEG-treated films were calculated using the relationship that κ is proportional to the square root of an elastic modulus [20,21]. The value reported for in-plane κ of Pristine film was 1.10 W/mK [22]. The sheet resistance (R_S_) was obtained with a four-point probe system to calculate the σ based on the equation σ = 1/(R_S_ × t), where t is the thickness of the film.

### 2.5. TE Humid Reliability Test

The size of the home-built humidity chamber using acryl plates was 300 mm × 300 mm × 300 mm (width × length × height). Relative humidity inside the chamber was controlled by a humidifier. After storage of Pristine, PEG solution-treated, PEG vapor-treated, glycerol solution-treated, and glycerol vapor-treated films in the humidity chamber (relative humidity 95% and temperature 27 °C), the S and R_S_ of the TE films were carefully re-measured to evaluate the TE reliability. To monitor TE humidity reliability, the values of S and R_S_ of films at different storage times were normalized to the initial value (0 h).

## 3. Results and Discussion

**Figure 1:**
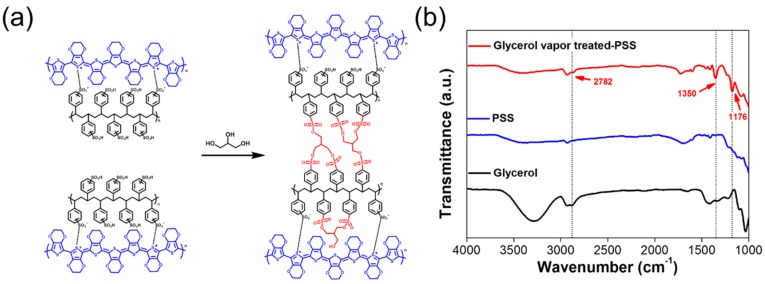
(**a**) Formation of sulfonic ester (covalent linkage) in intra and/or inter PSS. (**b**) FTIR spectra of neat PSS, glycerol vapor0treated PSS, and glycerol.

A glycerol has three hydroxyl groups (–OH) that harness hydrogen bonding and/or dipole-dipole- or dipole-charge-interaction with the sulfonic acid group (–SO_3_H) of PSS, resulting in higher affinity to PSS than PEDOT [6,23]. During glycerol treatment, the condensation reaction occurred between the sulfonic acid group (–SO_3_H) of PSS, and the hydroxyl group (–OH) of glycerol produces a sulfonic ester (S–O–C) group as the formation of the covalent linkage (Figure 1a) [6]. Thus, there are two types of chemical groups that exist in the PSS, namely, sulfonic acid and sulfonic ester. The hydroxyl groups of glycerol are eliminated during the crosslinking reaction, and the corresponding film shows little change in the total amount of oxygen atoms after the glycerol treatment (Table 1). It should be noted that the change om the number of oxygen atoms in the film significantly effects stability under humid conditions [13,14]. Similarly, increased ether groups in PEDOT:PSS film facilitate hydrogen bonds with water molecules, leading to electrical degradation of the film under a humid environment [13]. Hence, we expect that the glycerol treatment increases the humid reliability of the PEDOT:PSS TE film.

In order to verify the formation of a covalent linkage between the sulfonic acid of PSS and glycerol, we measured the FTIR spectra of neat PSS, glycerol vapor-treated PSS, and glycerol. After the treatment, a new peak at 2782 cm^−1^ appeared, which corresponds to the C-H vibration of modified PSS by the glycerol vapor (Figure 1b). Moreover, two peaks at 1180 and 1350 cm^−1^ arose, which are consistent with vibrations of the aromatic sulfonate ester group (S–O–C) [9,24,25]. These changes prove the formation of a covalent linkage between the sulfonic acid and the hydroxyl group. It is presumed that glycerol vapor is deposited on the PSS films and reacts with the sulfonic acid of PSS. In addition, the peak of 1352 cm^−1^ in the glycerol solution-treated PSS indicated the presence of aromatic sulfonate ester, as shown in Figure S2.

To observe the change of oxygen content in the films, XPS analysis was carried out. As shown in Table 1, the atomic percentage of O1s of the resultant PEDOT:PSS films through the solution and vapor PEG treatments is higher than that of Pristine film. In the glycerol treatment case, however, similar values are shown compared to Pristine film, implying little change of oxygen content in the corresponding PEDOT: PSS film. Thus, we demonstrated that the oxygen atoms in glycerol molecules do not affect the stability of PEDOT: PSS under humid conditions, and additive covalent bonds have a positive effect on TE reliability. 

**Figure 2:**
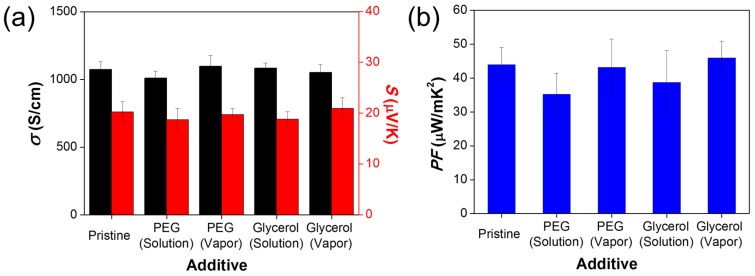
(**a**) σ and S, and (**b**) PF for Pristine, PEG solution-treated, PEG vapor-treated, glycerol solution-treated, and glycerol vapor-treated films.

We evaluated the average TE properties for Pristine, PEG solution-treated, PEG vapor-treated, glycerol solution-treated, and glycerol vapor-treated films. PEG is a typical crosslinking polymeric agent for PEDOT:PSS [5,8,9] The PEG-treated films were prepared to compare TE reliability with glycerol-treated films in a humid environment. As shown in Figure 2a, σ of the PEG solution-treated films showed the lowest value, and S of the glycerol vapor-treated films showed the highest value among them. Figure 2b shows that the PF of the glycerol-treated films was higher than that of the PEG-treated films, in the same treatment process. Accordingly, glycerol vapor-treated films had the highest PF of 45.95 ± 4.91 μW/mK^2^, which is a positive aspect of the ZT value.

**Figure 3:**
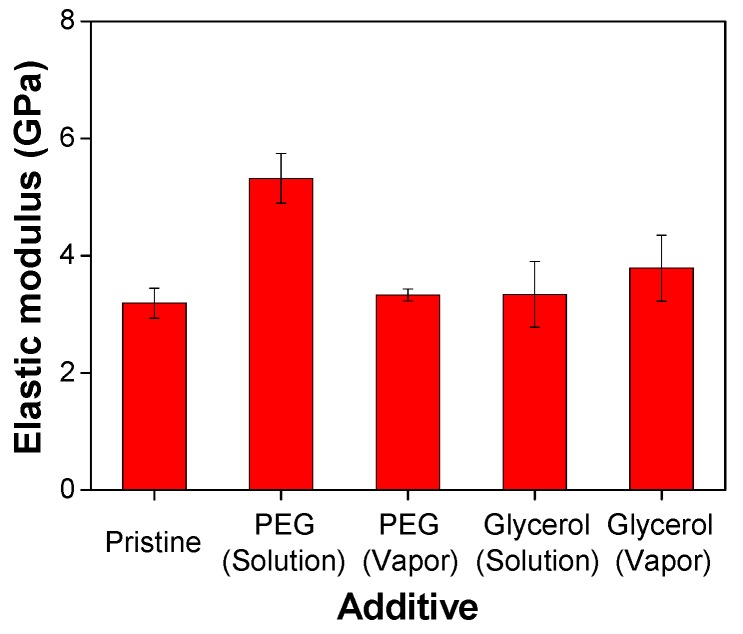
Elastic modulus of Pristine, PEG solution-treated, PEG vapor-treated, glycerol solution-treated, and glycerol vapor-treated films.

In-plane κ of a thin film with nanoscale thickness is difficult to measure because of heat loss to the substrate and the difficulty of free-standing film [26]. Therefore, we can estimate in-plane κ of the film based on the relationship between κ and the mechanical property of the film; κ is proportional to the square root of an elastic modulus [20,21]. We obtained the elastic modulus of the thin films using nanoindentation experiment in the tapping mode of an AFM [27]. The average values of elastic modulus were 3.2 ± 0.3, 5.3 ± 0.4, 3.3 ± 0.1, 3.4 ± 0.5, and 3.8 ± 0.6 GPa for Pristine, PEG solution-treated, PEG vapor-treated, glycerol solution-treated, and glycerol vapor-treated films, respectively (Figure 3). The origin of the enhancement of the elastic modulus of the PEG-treated and glycerol-treated films is related to the crosslinking reaction between crosslinking additives and PEDOT:PSS [5]. The higher elastic modulus of PEG solution-treated film was ascribed to the higher molecular weight of PEG compared to glycerol, which is a negative aspect of the ZT value [28]. Electronic contribution to thermal conductivity (κ_e_ = LσT, where L is the Lorenz number with a value of 2.44 × 10^−8^ W/ΩK^2^) for the five different films is similar. In this regard, the in-plane κ of PEG solution-treated, PEG vapor-treated, glycerol solution-treated, and glycerol vapor-treated films were 1.42, 1.12, 1.13, and 1.20 W/mK, respectively, which correspond to the ZT of approximately 0.74, 1.14, 1.02, and 1.15 × 10^−2^ at 300 K. In terms of in-plane κ with solution treatment, the PEG-treated film had a higher value than the glycerol film. However, with vapor treatment, the in-plane κ of PEG film was smaller than that of the glycerol film. Consequently, the ZT of glycerol solution-treated film was higher than that of PEG solution-treated film. In the vapor treatment, the ZT according to glycerol and PEG was similar, with higher values than those in the solution treatment. In terms of reliability, glycerol was better than PEG because of relatively higher elastic modulus and lower oxygen content (vide infra).

**Figure 4:**
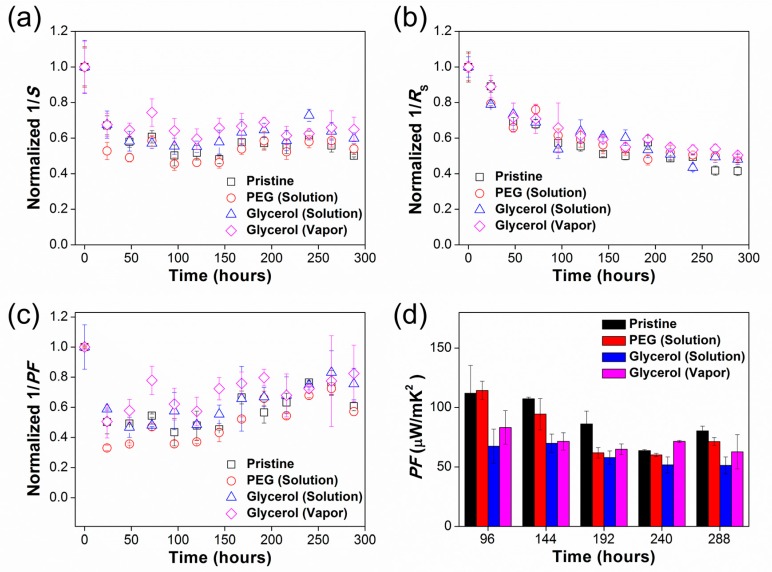
The normalized average value of (**a**) 1/S, (**b**) 1/R_S_, (**c**) 1/PF, and (**d**) PF for Pristine, PEG solution-treated, PEG vapor-treated, glycerol solution-treated, and glycerol vapor-treated films as a function of storage time. The humidity condition is relative humidity of 95% and temperature of 27 °C.

We evaluated the reliability of TE films by measuring S and R_S_ for Pristine, PEG solution-treated, PEG vapor-treated, glycerol solution-treated, and glycerol vapor-treated films, as a function of storage time in the home-made humidity chamber. As shown in Figure S3, the PEG vapor-treated film was easily washed out in the humid environment, so we were unable to evaluate the TE humidity reliability of the film. This film deformation can be explained by the increase in the hydrophilicity, which decreased the contact angle of the PEG vapor-treated film (27.72 ± 5.02°) compared to Pristine film (30.14 ± 0.50°), showing the affinity to water molecules. Figure 4a shows that the normalized average values of 1/S in the TE films are little changed within the error range of 15% according to the time increase, while those of 1/R_S_ decrease as the storage time increases in Figure 4b [9]. The normalized values were calculated by the true values of the measurement in Table S1. The glycerol solution-treated and vapor-treated films exhibit higher values of the normalized 1/S and 1/R_S_ than those of PEG solution-treated films. This tendency is consistent with the TE performance trend of chemically dedoped PEDOT:PSS TE films in the literature [29,30], and is attributed to the dedoping process of PEDOT molecules by the adsorbed water [15,21,31]. After 312 h, we were unable to obtain the normalized values of the TE characteristics (Figure S4). As shown in Figure S4, the Rs increases and S abruptly decreases after 312 h of storage compared to the values at 288 h. We infer that the delamination of Au electrodes from the active material results in an unusual TE performance concerning the reliability of the film. Thus, we show the TE performance until 288 h for systematical study of the reliability according to the storage time in Figure 4. After 288 h storage in the humid environment, the TE reliability (normalized 1/PF) of glycerol solution-treated film remained at 75% of its initial absolute 1/PF, while that of PEG solution-treated film reduced by 57% of its initial absolute 1/PF. Furthermore, the glycerol vapor treatment enabled the film to exhibit stronger TE humidity reliability than the glycerol solution-treated film, showing retention of 82% of its absolute 1/PF under the same conditions. Table S2 shows that the relative change in the PF of the glycerol treated-films are smaller than those in Pristine and PEG solution-treated films, indicating that glycerol yields a higher humidity reliability for the TE films compared to PEG. Furthermore, the glycerol vapor treatment allows the highest reliability of PEDOT: PSS TE film in a humid environment (Figure 4d, Figure S4, and Table S2). The PF is strongly dependent on the S at the initial storage time (0 h), according to the function PF=S^2^σ, where σ is proportional to 1/R_s_ [32,33,34]. In this relationship, S shows little change, and 1/R_s_ decreases as the storage time increases, as shown in Figure 4a,b, R3(a), and R3(b). That is, the relative change in the glycerol treatment is smaller than in the PEG treatment and no treatment as a control (Pristine film), increasing the relative reliability of the TE film with consistent TE performance. These observations support the view that glycerol is a better additive to improve TE humidity reliability than PEG [8,9,13] in organic TE devices.

**Figure 5:**
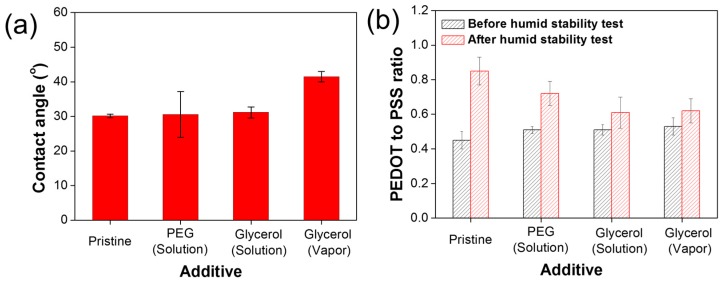
Average values of (**a**) contact angles and (**b**) PEDOT to PSS ratios of Pristine, PEG solution-treated, glycerol solution-treated, and glycerol vapor-treated films; before (0 h) and after humid stability test (288 h).

To get insight into the enhanced TE humidity reliability of the glycerol-treated films, we evaluated the hydrophobicity of the four different films using a contact angle measurement. As shown in Figure 5a, Pristine, PEG solution-treated, glycerol solution-treated, and glycerol vapor-treated films exhibited contact angles for water of 30.1 ± 0.5°, 30.6 ± 6.6°, 31.2 ± 1.6°, and 41.5 ± 1.5°, respectively, which indicates the formation degree of the covalent bond and shows the hydrophobicity according to the films. It is noted that the contact angle of the PEG solution-treated films showed larger standard variation than those of glycerol solution-treated and vapor-treated films. A possible explanation might be that ether groups in the PEG solution-treated film harness hydrogen bonds with water molecules when the ether groups do not participate in the condensation reaction [8,9]. Thus, we observed a larger atomic percentage of O1s of the PEG solution-treated film compared to that of the Pristine film, which is due to the existence of the ether groups of PEG. In the case of the glycerol treatment, three hydroxyl groups of glycerol molecules can readily react with the sulfonic acid of PSS with little change in the number of oxygen atoms in PEDOT:PSS film, resulting in higher hydrophobicity compared to the PEG-treated film [16] Additionally, the glycerol vapor treatment allowed the PEDOT:PSS film to have higher hydrophobicity than solution treatment, which explains the higher crosslinking density of glycerol vapor-treated film. This phenomenon was verified by the experimental results of elastic modulus and contact angle for glycerol vapor-treated film (Figure 3 and Figure 5a). Moreover, the slightly larger oxygen content in glycerol solution-treated film compared to that in vapor-treated film also indicates the lower TE humidity reliability.

To further understand the effect of glycerol treatment on the TE humidity reliability of the PEDOT:PSS films, we analyzed the change in the chemical composition for the four different films before and after a humidity stability test. PSS is hygroscopic and facilitates hydrogen bonds with water molecules, resulting in a change of chemical composition of PEDOT:PSS [35,36,37] As shown in Figure 5b and Figure S5, the PEDOT to PSS ratio of Pristine, PEG solution-treated, glycerol solution-treated, and glycerol vapor-treated films increased from 0.45 ± 0.05, 0.51 ± 0.02, 0.51 ± 0.03, and 0.53 ± 0.05, to 0.85 ± 0.08, 0.72 ± 0.07, 0.61 ± 0.09, and 0.62 ± 0.07, respectively, after the humidity stability test. The PEDOT to PSS ratios of glycerol vapor-treated film showed the lowest increase of 1.17 × 10^2^% compared to the original value (before the humidity stability test). On the contrary, the increased ratio of PEG solution-treated film was 1.41 × 10^2^%. Generally, the increase in PEDOT to PSS ratio increases σ [32,33,34]. The electrical properties of the films decrease even though the ratio increases after the stability test (Figure 4b). We infer that blocking electrical pathways causes this phenomenon, which can be attributed to dedoping of PEDOT, oxidation, structural change, etc. [38,39,40]. From these data, we deduced that the enhanced hydrophobicity of glycerol vapor-treated film can maintain the chemical composition of PEDOT:PSS against water molecules, resulting in the highest reliability of the TE film among the four different films in a humid environment. 

## 4. Conclusions

We fabricated a PEDOT:PSS TE film with superior reliability through glycerol vapor treatment, which was compared to non-treated (Pristine), PEG solution-treated, and glycerol solution-treated processes. The glycerol additive retained the TE performance for 288 h with 82% of initial absolute value (0 h), compared to 57% for PEG treatment under high humidity conditions (95% relative humidity, 27 °C temperature). We deduce that (1) low oxygen content in glycerol limits water adsorption, (2) the relative degree of crosslinking is improved, and (3) the corresponding relative ratio between PEDOT and PSS is preserved. The developed TE film can be utilized in a rainy outdoor environment for power generation, and in encapsulation of organic electronics for water protection.

## Figures and Tables

**Table 1:** Atomic percentage of different elements from XPS results on Pristine, PEG solution-treated, PEG vapor-treated, glycerol solution-treated, and glycerol vapor-treated films.

Sample	Atomic Percentage (%)		
	C1s	O1s	S2p
Pristine film	62.93 ± 3.21	23.27 ± 1.81	9.94 ± 1.19
PEG solution-treated film	67.19 ± 5.04	27.95 ± 1.12	3.21 ± 0.59
PEG vapor-treated film	66.52 ± 2.59	27.36 ± 1.04	3.73 ± 0.67
Glycerol solution-treated film	62.87 ± 3.46	25.27 ± 0.61	6.72 ± 1.41
Glycerol vapor-treated film	62.83 ± 3.02	24.05 ± 0.55	9.40 ± 1.60

## Supplementary Materials

The following are available online at https://www.mdpi.com/2079-4991/9/11/1591/s1, Figure S1: (a) Photograph of the Pristine film, (b) Schematic illustration for TE measurement; Figure S2: FT-IR spectra of neat PSS and glycerol solution treated PSS; Figure S3: A photograph of the PEG vapor treated film after storage in the humid chamber for 5 min; Figure S4: (a) R_s_, (b) S, and (c) PF for Pristine, PEG solution, glycerol solution, and glycerol vapor treated films as function of storage time. The humid condition is relative humidity with 95% and temperature with 27 °C; Figure S5: XPS spectra of the PEDOT:PSS films before and after humid stability test. (a) Pristine-, (b) PEG solution-treated, (c) Glycerol solution-treated, and (d) Glycerol vapor treat-films; Table S1: R_S_ and S in the Pristine, PEG solution-treated, glycerol solution-treated, and glycerol vapor treated-films according to storage time under high humid environment; Table S2: Degree of PF change in Pristine, PEG solution-treated, glycerol solution-treated, and glycerol vapor treated-films according to the storage time under a high humid environment.
