# Supplementary material for: Enhanced Humid Reliability of Organic Thermoelectrics via Crosslinking with Glycerol
Source: Nanomaterials (Basel). 2019 Nov 9;9(11):1591. doi: 10.3390/nano9111591 (PMC6915656; doi:10.3390/nano9111591)
Supplement: Supplementary file 1 [file nanomaterials-09-01591-s001.pdf]

nanomaterials

# Supplementary Materials

# Enhanced Humid Reliability of Organic Thermoelectrics via Crosslinking with Glycerol

**Jaeyun Kim [1,†], Jae Gyu Jang [2,†], Jeonghun Kwak [3,*], Jong-In Hong [2,*] and Sung Hyun Kim [4,*]**

[1] School of Electrical and Computer Engineering, University of Seoul, Seoul 02504, Korea; kimjaeyun24@naver.com
[2] Department of Chemistry, Seoul National University, Seoul 08826, Korea; jangjaegyu@snu.ac.kr
[3] Department of Electrical and Computer Engineering, Inter-university Semiconductor Research Center, Seoul National University, Seoul 08826, Korea
[4] Department of Carbon Convergence Engineering, Wonkwang Univeristy, Iksan 54538, Korea
* Correspondence: jkwak@snu.ac.kr (J.K.); jihong@snu.ac.kr (J.-I.H.); shkim75@wku.ac.kr (S.H.K.); Tel.: +82-02-880-1782 (J.K.); +82-02-874-5902 (J.-I.H.); +82-063-850-6393 (S.H.K.)
† These authors contributed equally to this work.

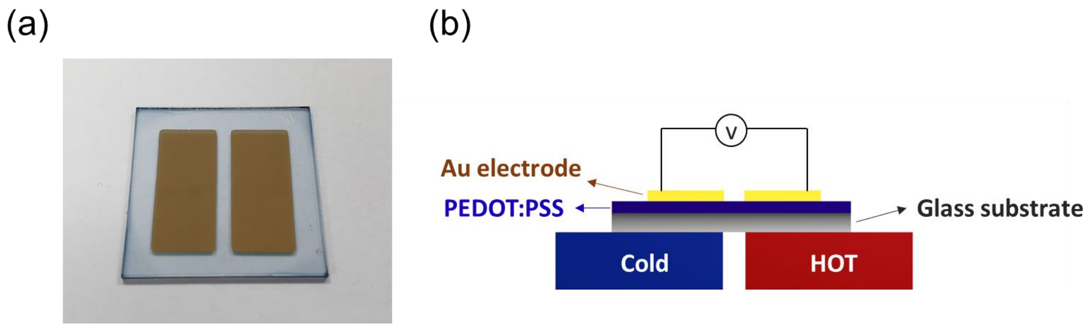


**Figure S1.** (**a**) Photograph of the Pristine film, (**b**) Schematic illustration for TE measurement.

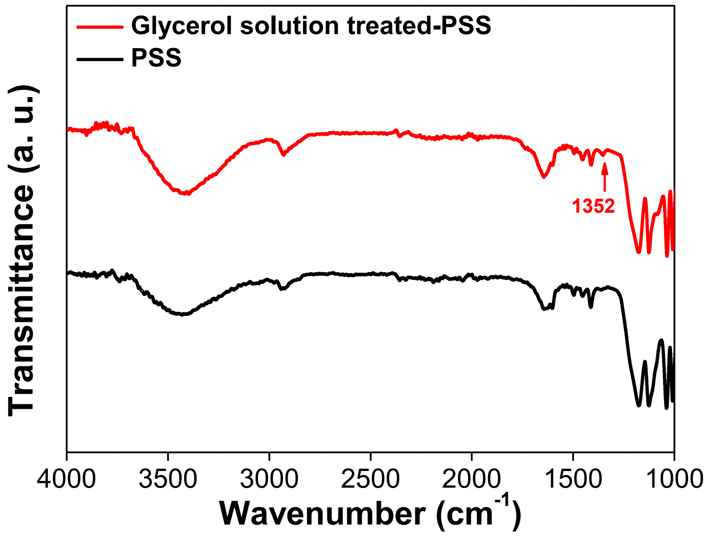


**Figure S2.** FT-IR spectra of neat PSS and glycerol solution treated PSS.

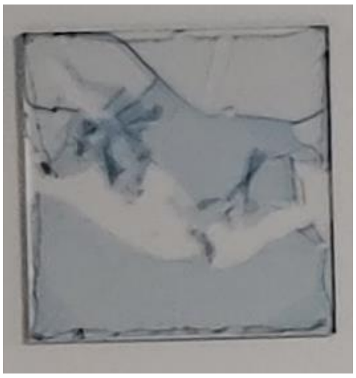

**Figure S3.** A photograph of the PEG vapor treated film after storage in humid chamber for 5 min.

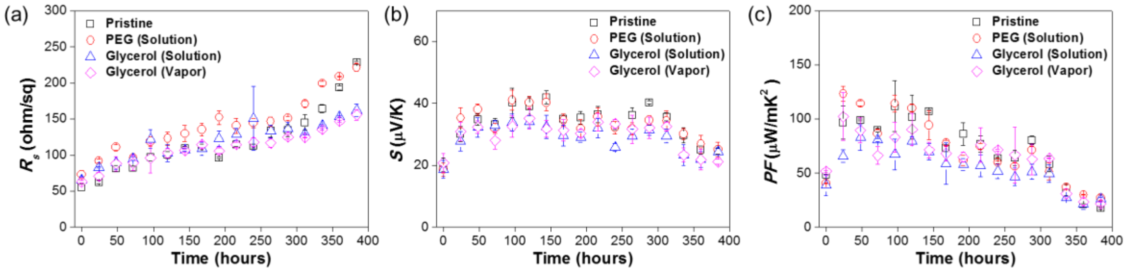


**Figure S4.** (**a**) $R_s$, (**b**) *S*, and (**c**) *PF* for Pristine-, PEG solution treated-, glycerol solution treated-, and glycerol vapor treated-film as function of storage time. The humid condition is a relative humidity with 95% and temperature with 27 °C.

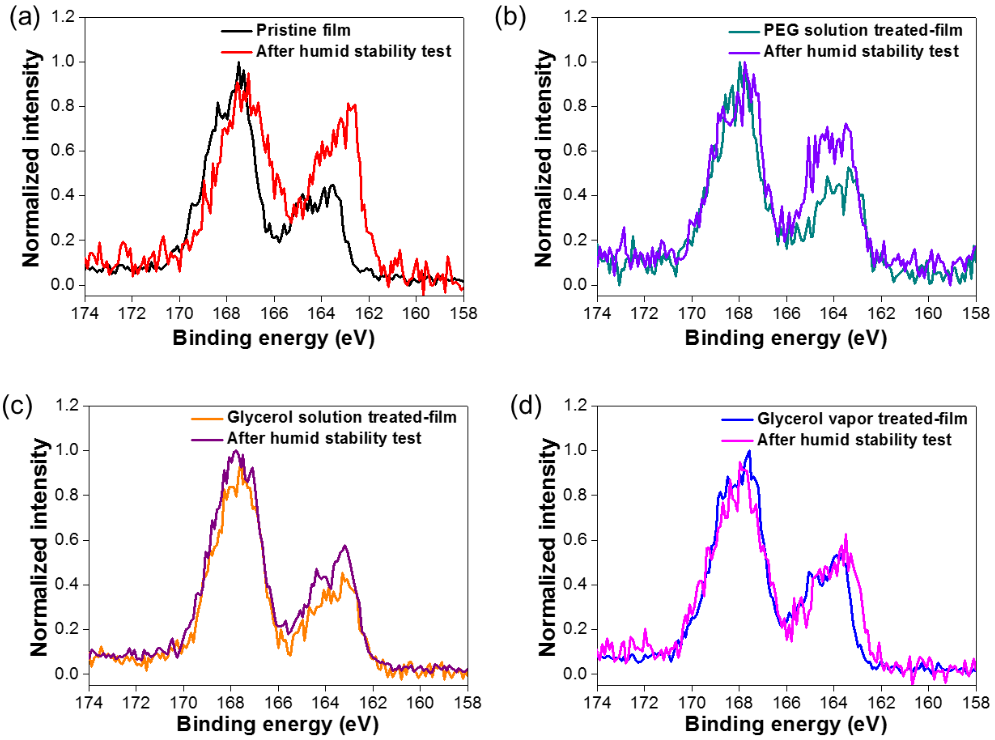


**Figure S5.** XPS spectra of the PEDOT:PSS films before and after humid stability test. (a) Pristine-, (b) PEG solution treated-, (c) Glycerol solution treated-, and (d) Glycerol vapor treat-film.

**Table S1.** *Rs and S* in the Pristine, PEG solution treated-, glycerol solution treated-, and glycerol vapor treated-films according to storage time under high humid environment.

| Storage time (hours) | Sample | | | | | | | |
|---|---|---|---|---|---|---|---|---|
| | Pristine film | | PEG solution treated film | | Glycerol solution treated film | | Glycerol vapor treated film | |
| | *R*s (ohm/sq) | *S* (μV/K) | *R*s (ohm/sq) | *S* (μV/K) | *R*s (ohm/sq) | *S* (μV/K) | *R*s (ohm/sq) | *S* (μV/K) |
| 0 | 55.64 ± 4.72 | 20.22 ± 2.16 | 72.96 ± 5.55 | 18.66 ± 2.13 | 65.60 ± 4.18 | 18.80 ± 2.81 | 63.04 ± 1.95 | 20.90 ± 3.03 |
| 24 | 62.46 ± 2.34 | 30.11 ± 2.41 | 92.01 ± 2.58 | 35.36 ± 3.26 | 83.14 ± 0.82 | 27.82 ± 3.14 | 70.84 ± 5.04 | 30.97 ± 2.98 |
| 48 | 81.51 ± 3.87 | 34.90 ± 1.84 | 110.85 ± 3.99 | 38.06 ± 1.90 | 89.31 ± 8.41 | 32.27 ± 3.07 | 88.15 ± 10.11 | 32.45 ± 2.02 |
| 72 | 82.05 ± 3.05 | 33.28 ± 1.91 | 95.98 ± 3.32 | 31.91 ± 1.62 | 95.26 ± 2.58 | 32.94 ± 1.61 | 89.01 ± 10.31 | 28.09 ± 2.91 |
| 96 | 96.99 ± 4.97 | 40.23 ± 4.73 | 118.75 ± 7.09 | 41.09 ± 3.21 | 122.11 ± 13.19 | 33.91 ± 2.79 | 95.94 ± 20.52 | 32.59 ± 3.51 |
| 120 | 100.46 ± 4.80 | 39.07 ± 3.07 | 122.97 ± 9.23 | 40.28 ± 1.96 | 102.84 ± 11.67 | 34.07 ± 1.96 | 102.72 ± 6.57 | 35.14 ± 3.41 |
| 144 | 109.12 ± 3.06 | 42.01 ± 2.10 | 129.79 ± 11.41 | 40.33 ± 2.77 | 107.28 ± 1.87 | 32.52 ± 3.78 | 106.18 ± 2.45 | 31.85 ± 2.73 |
| 168 | 111.28 ± 3.90 | 35.06 ± 1.20 | 135.31 ± 8.80 | 34.63 ± 1.97 | 108.90 ± 8.90 | 29.71 ± 3.32 | 114.69 ± 4.45 | 31.35 ± 3.45 |
| 192 | 96.56 ± 1.07 | 35.36 ± 2.03 | 152.09 ± 9.62 | 31.91 ± 2.85 | 122.86 ± 11.12 | 29.12 ± 1.69 | 106.04 ± 3.86 | 30.35 ± 1.22 |
| 216 | 114.46 ± 3.06 | 36.45 ± 2.70 | 140.73 ± 10.59 | 35.70 ± 2.90 | 128.82 ± 8.56 | 32.09 ± 3.12 | 114.69 ± 3.57 | 34.03 ± 2.86 |
| 240 | 112.58 ± 2.54 | 32.90 ± 1.34 | 144.41 ± 6.28 | 32.17 ± 1.94 | 151.01 ± 44.12 | 25.83 ± 1.22 | 117.83 ± 5.90 | 33.44 ± 1.47 |
| 264 | 133.22 ± 8.36 | 36.21 ± 2.40 | 146.35 ± 6.31 | 31.86 ± 1.95 | 132.79 ± 8.97 | 29.46 ± 2.27 | 116.86 ± 5.68 | 31.70 ± 4.53 |
| 288 | 134.23 ± 8.23 | 40.33 ± 0.54 | 150.90 ± 2.83 | 34.60 ± 1.45 | 136.49 ± 8.94 | 31.39 ± 1.59 | 125.08 ± 2.18 | 32.21 ± 3.40 |

**Table S2.** Degree of *PF* change in the Pristine-, PEG solution treated-, glycerol solution treated-, and glycerol vapor treated-films according to the storage time under a high humid environment.

| **Storage time (hours)** | **Sample** | | | | | | | |
|---|---|---|---|---|---|---|---|---|
| | **Pristine film** | | **PEG solution treated film** | | **Glycerol solution treated film** | | **Glycerol vapor treated film** | |
| | **PF (μW/mK$^2$)** | **Degree of change*** | **PF (μW/mK$^2$)** | **Degree of change** | **PF (μW/mK$^2$)** | **Degree of change** | **PF (μW/mK$^2$)** | **Degree of change** |
| 0 | 48.70 ± 0.38 | - | 40.74 ± 0.17 | - | 38.68 ± 9.57 | - | 51.67 ± 0.92 | - |
| 24 | 96.83 ± 15.18 | 99% | 123.33 ± 6.86 | 203% | 65.81 ± 5.76 | 70% | 102.18 ± 22.08 | 98% |
| 48 | 99.03 ± 0.81 | 103% | 114.36 ± 2.35 | 181% | 82.84 ± 11.88 | 114% | 89.47 ± 11.67 | 73% |
| 72 | 89.49 ± 1.82 | 84% | 86.49 ± 4.45 | 112% | 80.51 ± 1.29 | 108% | 66.37 ± 7.99 | 28% |
| 96 | 111.87 ± 23.50 | 130% | 114.18 ± 7.69 | 180% | 67.34 ± 14.41 | 74% | 83.19 ± 14.06 | 61% |
| 120 | 101.77 ±2 0.36 | 109% | 110.07 ± 3.03 | 170% | 79.80 ± 3.84 | 106% | 90.29 ± 14.78 | 75% |
| 144 | 107.22 ± 1.33 | 120% | 94.31 ± 13.11 | 131% | 69.89 ± 7.74 | 81% | 71.45 ± 7.29 | 38% |
| 168 | 73.24 ± 3.27 | 50% | 78.12 ± 2.18 | 92% | 58.89 ± 19.24 | 52% | 68.16 ± 6.86 | 32% |
| 192 | 86.15 ± 10.66 | 77% | 61.80 ± 4.43 | 52% | 57.93 ± 5.53 | 50% | 64.88 ± 4.49 | 26% |
| 216 | 77.11 ± 7.51 | 58% | 75.03 ± 2.61 | 84% | 56.96 ± 10.22 | 47% | 76.15 ±1 5.96 | 47% |
| 240 | 63.73 ± 0.81 | 31% | 60.09 ± 1.35 | 47% | 51.76 ± 6.68 | 34% | 71.45 ± 0.81 | 38% |
| 264 | 64.03 ± 6.94 | 31% | 56.29 ± 3.16 | 38% | 46.56 ± 8.10 | 20% | 66.75 ± 25.98 | 29% |
| 288 | 80.37 ± 3.92 | 65% | 71.34 ± 3.35 | 75% | 51.28 ± 7.14 | 33% | 62.71 ± 14.35 | 21% |

*Degree of change (%) = ($PF_{at\ 0\ hour}$ – $PF_{at\ storage\ time}$)/ $PF_{at\ 0\ hour}$ × 100
